# Scanning probe microscopy and related methods

**DOI:** 10.3762/bjnano.1.18

**Published:** 2010-12-22

**Authors:** Ernst Meyer

**Affiliations:** 1 Department of Physics, University of Basel, Klingelbergstr. 82, CH-4056 Basel

Since the invention of scanning tunnelling microscopy (STM) [1] and atomic force microscopy (AFM) [2], a new class of local probe microscopes has entered the laboratories around the world. Scanning probe microscopy (SPM) uses probing tips to map properties, such as topography, local adhesive forces, elasticity, friction or magnetic properties. In the emerging fields of nanoscience and nanotechnology these types of microscopes help to characterize the nanoworld. In addition, local probes can also be used to modify the surfaces and to perform lithography processes.

AFM, especially, has turned out to be a versatile instrument, which can be operated in various environments, such as liquids, gases or vacuum. High and low temperature versions are available, which allow scientists to explore a large variety of materials. The strategy is not to prepare samples according to the requirements of the microscopy technique, but to perform experiments in its most native state, e.g., to study biological material in liquids. Questions to be addressed originate from almost all scientific areas. One example is the field of molecular electronics, where single molecules are investigated in order to perform specific tasks, e.g., molecular switches, molecular transistors or even molecular processors. In this area, STM and AFM have become essential tools to characterize the structure and function of molecules on surfaces.

AFM has evolved considerably in the last few years, where new operation modes, such as non-contact force microscopy (nc-AFM), Kelvin probe force microscopy (KPFM) or friction force microscopy (FFM), were developed. One main focus is the high resolution capabilities of nc-AFM, which were drastically improved. Atomic resolution on metals, semiconductors [3] and insulators was achieved. Recently, the atomic structure of single molecules was identified by nc-AFM, which gives new opportunities to investigate the local structure of these molecules [4]. In this Thematic Series, the structure of oxides is explored by the combination of nc-AFM. Colour centres are characterized by KPFM and tunnelling spectroscopy. The arrangement of molecules on insulators is another type of application, which is discussed in the present Thematic Series. The ability to measure across phase transitions gives insight into fascinating phenomena, such as metal-superconductor transitions or metal-insulator transitions.

Another important development is related to nanomechanics, where phenomena, such as friction, wear, elasticity and plasticity are studied on an atomic scale. Atomic friction has been studied in great detail, where the main mechanism is related to atomic instabilities, which lead to the characteristic stick slip behaviour. The loading and velocitiy dependence were interpreted in terms of a thermally activated Prandtl–Tomlinson-model [5–6]. The transition into the superlubricity regime was observed for incommensurate contacts [7] and for contacts at low loads [8]. Furthermore, the control of atomic friction was achieved by electrostatic and mechanical actuation of the nanoscale contacts [8]. In this Thematic Series, a microfabricated tribometer bridges the gap between the nanometer-scale to the micron scale of micromachinery. Mechanical actuation is used to reduce friction of these micro contacts.

An important aspect of SPM is the possibility to modify surfaces. The probing tip can be either used to push or pull atoms, molecules or particles across surfaces. These experiments give information about the local bonding and to explore friction and wear mechanisms. Two different regimes were observed, which were related to the commensurability of the contacts [9]. The manipulation of a large number of particles gives also access to the size and shapes of the particles [10] and is discussed in this Thematic Series as well.

**Figure 1:**
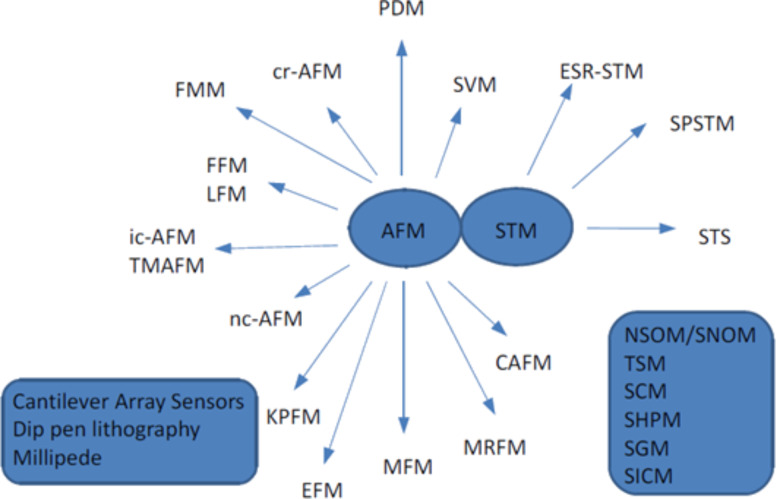
Scanning probe microscopy: A large familiy of microscopes, which have in common that they use local probes to characterize surfaces. AFM: Atomic Force MicroscopySTM: Scanning Tunneling Microscopy, PDM: Phase Detection Microscopy, FMM: Force Modulation Microscopy, ic-AFM: intermittent contact AFM, TMAFM: tapping mode AFM, nc-AFM: non-contact AFM, KPFM: Kelvin probe force microscopy, EFM: Electrostatic force microscopy, MFM: Magnetic force microscopy, MRFM: Magnetic resonance force microscopy, NSOM: Near-field scanning optical microscopy, SNOM: Scanning nearfield optical microscopy, TSM: Thermal scanning microscopy, cr-AFM: contact-resonance AFM, SPSTM: Spin polarized STM, SHPM: Scanning Hall probe microscopy, SGM: Scanning gate microscopy, SVM: Scanning voltage microscopy / Nanopotentiometry, ESR-STM: Electron spin resonance-STM, SICM: Scanning ion conductance microscopy, CAFM: Conductive AFM.

A schematic drawing of the family of scanning probe microscopes in Figure 1 demonstrates how fast and diverse this field of research develops. I hope that this Thematic Series will help the reader to get insights in this fascinating world. Furthermore, I want to thank the colleagues for their excellent contributions.

Ernst Meyer

Basel, December 2010
